# Use of Homeostatic Model Assessment Indexes for the Identification of Metabolic Syndrome and Insulin Resistance among Cuban-Americans: A Cross Sectional Study

**DOI:** 10.9734/BJMMR/2014/8988

**Published:** 2014-06-30

**Authors:** Joel C. Exebio, Sahar Ajabshir, Gustavo G. Zarini, Joan Vaccaro, Fatma G. Huffman

**Affiliations:** 1 Florida International University, Robert Stempel School of Public Health and Social Work, Department of Dietetics and Nutrition, AHC I - 435, 11200 S. W. 8th Street, Miami, FL 33199, USA.

**Keywords:** Insulin resistance, metabolic syndrome, homeostatic model assessment, Cuban Americans

## Abstract

**Aim:**

to determine cut off points for The Homeostatic Model Assessment Index 1 and 2 (HOMA-1 and HOMA-2) for identifying insulin resistance and metabolic syndrome among a Cuban-American population.

**Study Design:**

Cross sectional.

**Place and Duration of Study:**

Florida International University, Robert Stempel School of Public Health and Social Work, Department of Dietetics and Nutrition, Miami, FL from July 2010 to December 2011.

**Methodology:**

Subjects without diabetes residing in South Florida were enrolled (N=146, aged 37 to 83 years). The HOMA1-IR and HOMA2-IR 90^th^ percentile in the healthy group (n=75) was used as the cut-off point for insulin resistance. A ROC curve was constructed to determine the cut-off point for metabolic syndrome.

**Results:**

HOMA1-IR was associated with BMI, central obesity, and triglycerides (*P*<0.05). HOMA2-IR was associated with BMI, central obesity, total cholesterol, HDL-cholesterol and LDL-cholesterol (*P*<0.05). The cut-off points for insulin resistance for HOMA-1 and HOMA-2 were >3.95 and >2.20 and for metabolic syndrome were >2.98 (63.4% sensitivity and 73.3% specificity) and >1.55 (60.6% sensitivity and 66.7% specificity), respectively.

**Conclusion:**

HOMA cut-off points may be used as a screening tool to identify insulin resistance and metabolic syndrome among Cuban-Americans living in South Florida.

## 1. INTRODUCTION

Insulin resistance (IR) is the decrease in the capacity of several tissues to respond to insulin, failing to transport glucose into the cell [1]. Metabolic syndrome (MS) is a condition that encompasses several metabolic disorders related to insulin resistance including abdominal obesity, hypertension, hyperlipidemia, low HDL cholesterol, and impaired fasting plasma glucose [2]. Both conditions are risk factors for type 2 diabetes (T2D) and cardiovascular disease and its prevalence has increased notably in the recent years [3,4].

The hyperinsulinemic euglycemic clamp is considered the gold standard to diagnose IR, however, it is not practical due to inconvenience, time and cost. The use of indirect methods that can be sensitive, accurate and replicable has been proposed [5].

The Homeostatic model assessment (HOMA) is a secondary measure of insulin resistance based on fasting plasma glucose and fasting insulin that has been validated against the hyperinsulinemic euglycemic clamp. The first version of HOMA1-IR was published in 1985 assuming a linear relationship in the feedback glucose-insulin. Even though the HOMA1-IR has been widely used in epidemiological research, it has been criticized for assuming this simplistic approach [6]. The second version of the model (HOMA2-IR) established a non-linear relationship that better reflect the real physiologic interaction among plasma glucose and insulin and corrects for peripheral and hepatic glucose resistance, as well as renal glucose loss, making this model appropriate for subjects with hyperglycemia [7].

Several studies have established population-specific cut-off points to identify insulin resistance and metabolic syndrome using the original HOMA1-IR index; however, cut-off values for HOMA2-IR are scarce [8–17]. Cut-off values are ethnic specific; therefore, they should be independently tested in high risk populations [18].

In order to evaluate HOMA cut-off points to detect MS, a standardized definition should be used. The International Diabetes Federation (IDF) has developed a definition of MS that unifies previous definitions proposed by different organizations and that is easy to apply in clinical settings and compare among different populations [19].

Cuban-Americans represent the third-largest minority group in the United States of who two thirds live in the state of Florida. Approximately 16% of Cuban-Americans ages 45–74 years in the US have diabetes which is 1.3 times higher than in non-Hispanic Whites [20]. Since IR is a risk factor for diabetes, indentifying subjects with IR or MS and providing adequate treatment may prevent the progression to T2D in Cuban-Americans.

The objective of the present study was to determine cut off points for HOMA-1 and HOMA-2 to identify insulin resistance and metabolic syndrome, and to compare the association of both indexes with individual metabolic syndrome components in a sample of Cuban-Americans.

## 2. MATERIALS AND METHODS

### 2.1 Participants

Male and female Cuban-Americans with and without T2D were included in a cross sectional study of risk factors for T2D and cardiovascular disease. Recruitment of participants was conducted in alternate phases of potential subjects with and without T2D, age matching subjects by age group. Individuals were initially recruited by random selection (every tenth address) from a randomly generated mailing list of Cuban-American subjects with and without T2D in Miami-Dade and Broward counties, Florida. Letters (10,000) in English and Spanish, including an invitation flyer, were sent using a pre-purchased list (5,000 with, 5,000 without T2D). The list was purchased from Knowledge Base Marketing Inc., Richardson, TX, 75081. Three hundred letters (3%) were returned due to wrong address and 388 (4%) responded. The inclusion criteria for subjects with T2D were self-reported Cuban or Cuban- American ethnicity; age ≥30 years; self-report of a medical diagnosis of T2D; able to understand and complete all of the study protocols in English or Spanish; and willing and able to read and sign an informed consent form. Exclusion criteria were; pregnant or lactating women, presence of any thyroid disorders and any major psychiatric disorders. Subjects without T2D were selected using the same inclusion/exclusion criteria with the exception of the previous diagnosis of T2D. Initial telephone interview determined previous diagnosis of diabetes, age and gender. Objective of the study was explained, and initial treatment modalities for diabetes were asked. Only 18 subjects did not qualify for the study; for not being Cuban-American (n=2), age <30 years old (n=9), and having any other chronic illnesses (n=7). Eligible participants were invited to participate in a morning session for blood draw and other study related data collection at the Human Nutrition Laboratory, Department of Dietetics and Nutrition, Florida International University. They were instructed to fast for at least 8 hours, avoid smoking and avoid any unusual physical activity prior to the morning session. For the present study only subjects without T2D were included because subjects with T2D were already taking medications that decrease their blood glucose and lipid values to normal levels.

Twenty subjects were excluded for having values of fasting plasma glucose outside the range for HOMA2-IR calculations (54.1 to 450.5 mg/dl) and seven outside the range for insulin (2.9 to 43.8mU/L). Total data was available for 146 Cuban-Americans without T2D. The study was approved by the Florida International University, Institutional Review board for the inclusion of human subjects.

### 2.2 Demographic Data

A socio demographic questionnaire was given to each participant to complete, which included questions related to age, gender, smoking, medication for diabetes, hypertension and cholesterol.

### 2.3 Anthropometric Measures

Height and weight were measured using a SECA 700M balance scale (SECA Corporation, Columbia, MD, US). Body mass index (BMI) was calculated as weight in kg/height in m^2^.

### 2.4 Blood Pressure

Blood pressure was measured twice in a sitting position after a 15 minute rest using a Omron automatic blood pressure monitor HEM-711DLX (Omron Corporation, Bannockburn, Illinois, US).

### 2.5 Blood Collection

Venous blood (20ml) was collected from each subject after an overnight fast. Blood samples were collected into a Vacutainer serum separator tube (SST) for analysis of lipids. After coagulation was completed the SST was centrifuged at 2500 RPM for 30 minutes. Lipid panel was assayed by enzymatic methods. Insulin levels were measured using a sandwich enzyme-linked immunosorbent assay ELISA C1052 (Millipore Corporation, Billerica, MA, US). The sensitivity range of this assay was 2–200μU/mL and the intra-assay and inter-assay coefficients of variations were 5.96%±1.17 and 10.3%±0.9, respectively. Glucose levels were measured by hexokinase enzymatic method.

The HOMA 1-IR index was calculated using the formula: HOMA1-IR=[fasting plasma insulin (μU/ml) × fasting plasma glucose (mmol/L)]/22.5. HOMA2-IR was calculated using the online calculator [21].

### 2.6 Definition of Metabolic Syndrome

Metabolic syndrome was defined according to the IDF criteria which includes abdominal obesity with specific cut-off values for men and women plus any two of the following four factors: raised triglyceride level (≥150 mg/dl) or taking medication, reduced HDL cholesterol (<40 mg/dl in males and <50 mg/dl in females) or taking cholesterol medication; raised blood pressure (systolic blood pressure ≥130 or diastolic blood pressure ≥85 mm Hg), or taking antihypertensive medication, elevated fasting plasma glucose (≥100 mg/dl) or previous diagnosis of T2D [19].

### 2.7 Statistical Analysis

The Kolmogorov-Smirnov test was applied to determine the normality of the data. Data that followed a normal distribution was reported as mean ± SD, otherwise, median (interquantile range) was reported. Since HOMA indexes were not normally distributed, the Mann-Whitney test was run to compare the healthy and metabolic syndrome groups. Normally distributed continues data were compared by t-test and categorical data by chi-square test among the healthy and metabolic syndrome groups. The mean values for BMI, waist circumference, triglycerides, total cholesterol, HDL-cholesterol, and LDL-cholesterol among the 1^st^ and 4^th^ quartiles of HOMA indexes were compared using t-test. The cut-off points for insulin resistance were based on the 90^th^ percentile of HOMA indexes for the healthy population. A receiver operating characteristic (ROC) analysis was conducted to determine sensitivity and specificity of HOMA indexes in detecting MS at different cut-off points. The ROC curve plots sensitivity versus 1 minus specificity at each cut-off level. The greater the area under the curve (AUC), the better the prediction value of HOMA indexes for detecting MS. An AUC of 0.5 means no prediction value, whereas an AUC of 1 means perfect prediction value. The optimal cut-off point was determined by the value that had the largest sum of sensitivity and specificity, along with sensitivity and specificity ≥60%. The Z statistic pairwise comparison was used to compare the AUCs. Statistical analysis was run using SPSS 18 (Chicago). A P-value <0.05 was considered significant.

## 3. RESULTS AND DISCUSSION

A total of 75 healthy subjects and 71 subjects with MS were included in the analysis. Subjects with metabolic syndrome were older (*P*=.03), had higher BMI (*P*<.001), higher waist circumference (*P*<.001), higher systolic blood pressure (*P*<.001), diastolic blood pressure (*P*<.001), higher triglycerides (*P*<.001), lower HDL cholesterol (*P*<.001), higher fasting plasma glucose (*P*<.001), higher insulin (*P*<.001), higher HOMA1-IR (*P*<.001) and higher HOMA2-IR (*P*<.001). No difference was observed for gender, total cholesterol and LDL cholesterol (Table 1).

**Fig. 1.**
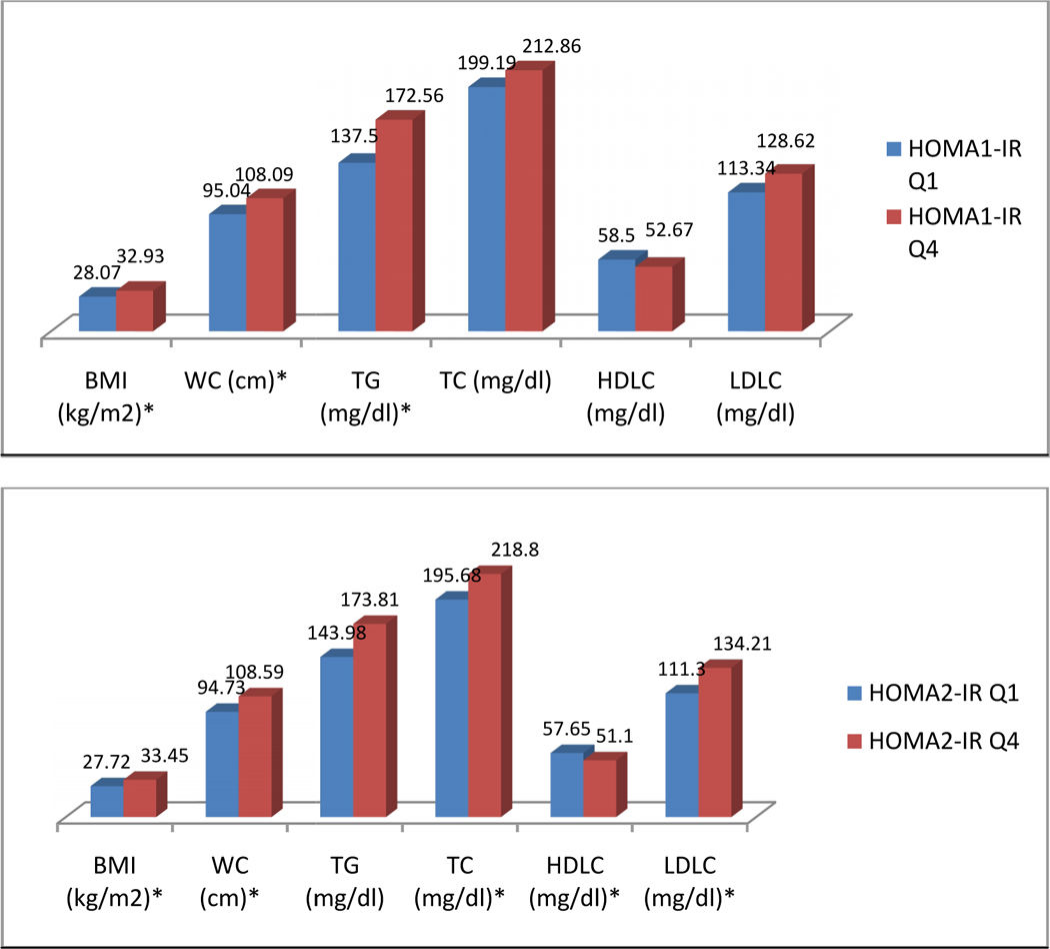
Comparison of clinical and metabolic characteristics according to HOMA-IR indexes quartiles in Cuban-Americans Q1=first quartile, Q4=fourth quartile, BMI=body mass index, WC=waist circumference, TG=triglyceride, TC=total cholesterol, HDLC=HDL-cholesterol, LDLC=LDL-cholesterol; t-test between the 1^s^ and 4^th^ quartiles. Data presented as means. *P<0.05

Fig. 1 compares the means of BMI, waist circumference and metabolic parameters among the 1^st^ and 4^th^ quartiles of HOMA indexes. For the HOMA1-IR the difference is significant only for BMI (*P*<.001), waist circumference (*P*<.001) and triglycerides (*P*<.001). For the HOMA2-IR index the difference is significant among BMI (*P*<.001), waist circumference (*P*<.001), total cholesterol (*P*=.03), HDL cholesterol (*P*=.03), and LDL cholesterol (*P*=0.02).

The cut-off points for insulin resistance for HOMA-1 and HOMA-2 were >3.95 and >2.20 and for metabolic syndrome were >2.98 (63.4% sensitivity and 73.3% specificity) and >1.55 (60.6% sensitivity and 66.7% specificity), respectively (Table 2).

The AUC for the prediction of MS using HOMA1-IR was 0.688 (95% CI: 0.622 to 0.753) and for HOMA2-IR was 0.806 (95% CI: 0.752 to 0.860). Both areas were statistically significant (*P*<.001). Both AUCs were significantly different (*P*=.03). HOMA2-IR showed a bigger AUC compared to HOMA1-IR.

The association among IR and development of T2D and cardiovascular disease has been clearly established [3]. However, identification of individuals with IR is still a controversial issue. This study investigated the optimal cut-off points for HOMA1-IR and HOMA2-IR to identify IR and MS in a sample of Cuban-Americans residing in South Florida.

Both indexes showed different associations with anthropometric and metabolic variables. Obesity is considered the main cause of insulin resistance and is highly related with other components of metabolic syndrome [22]. Both indexes were associated with BMI and waist circumference. Subjects with higher values for HOMA indexes showed greater obesity measures. In contrast, the association among each index and metabolic variables was not consistent. HOMA1-IR was associated only with triglycerides and HOMA2-IR was associated with total cholesterol, HDL and LDL cholesterol. These results may indicate that different physiological mechanisms are represented differently in each HOMA model. For instance, the HOMA1-IR index was calibrated against an insulin assay used in the 1970s which underestimates insulin sensitivity compared to later assays [18]. In fact, the HOMA2-IR has been calibrated against current assays which incorporate estimates of pro-insulin secretion [18]. In addition, the insulin glucose feedback mechanism was assumed to be linear in the original index. The HOMA2-IR reflects a more accurate no linear relationship among insulin and glucose which represents better the physiological reality. In addition, HOMA2-IR considers the variations in hepatic and peripheral insulin resistance [10]. Safar et al. [16] suggested that the difference between indexes is greater at higher levels of fasting insulin, meaning that HOMA1-IR scores increase more than HOMA2-IR scores under these conditions.

In our population, mean insulin values were highly elevated in both the healthy and MS groups (10.41±5.01mU/L and 14.04±6.27mU/L, respectively). This panorama may reflect a higher risk for IR and MS even among healthy subjects in this sample of Cuban-Americans. In fact, the optimal HOMA1-IR cut off point for IR was >3.95. This score is higher than found in other populations. A cut-off value of 2.71 has been found in a multiethnic sample of Brazilians [8]. Similarly a score of 2.9 have been reported for Kuwaiti women and 2.5 for the general Kuwaiti population [16,17]. In addition, Buccini et al. [10] determined a cut-off point of 2.64 for Argentineans. Bonora et al. [11] found a cut-off value of 2.77 in Italians and Yeni-Komshian et al. [12] 2.7 in North-Americans. On the other hand, a study conducted in a Spanish population found a cut-off value of 3.8 [13]. The variation may be due to genetic factors and different methods used for the calculation of cut-off points for the detection of IR. Some studies have used the 75^th^ percentile, while other has used the 90^th^ percentile of HOMA1-IR as the cut-off point to identify IR.

Our sample was composed of White Cuban-Americans. White Cuban-Americans are Spanish descents. This may explain in part, why our cut-off point is closer to the one found in the Spanish population. Still our values are higher than found in any other population which may reflect a higher risk for IR among Cuban-Americans.

The optimal HOMA1-IR cut off point for detecting MS was >2.98. This point is lower than the one identified for IR using the same index. Since MS is a cluster of different metabolic abnormalities, it is expected that the cut-off point will be smaller than for IR alone. However, the cut-off point is still higher than found in previous studies. A cut-off value of 2.3 was identified in healthy Brazilians [9]. In Korean subjects without diabetes the optimal cut-off was 2.34 [14]. A value of 2.5 was identified in a small sample of Brazilian obese children [15].

Regarding HOMA2-IR, there is a scarcity of studies exploring this topic. Geloneze et al. [9] identified values of 1.8 and 1.4 for IR and MS in Brazilians. Similarly, Buccini et al. [10] proposed the value of 1.67 to detect IR in Argentineans using the HOMA2-IR index. The values identified in the present study were 2.20 for IR and 1.55 for MS, which are closer to the ones found in previous studies. This may reflect a smaller variability in HOMA2-IR among populations.

The ROC analysis showed that both AUCs (HOMA1-IR and HOMA2-IR) were statistically significant meaning that they are both associated with MS. However, the AUCs were also significantly different from each other. This means that subjects identified with MS using HOMA1-IR may be missed using HOMA2-IR affecting clinical decision making. This may explain in part, the inconsistency of cut-off points reported by previous studies. In addition, the different association that each index has with metabolic factors potentiates the idea that they differ in their ability as a diagnostic tool.

Limitations of our study include the cross sectional design which provides only one measure of fasting plasma glucose and insulin. Both are highly variable and ideally two samples of both are necessary to obtain a more accurate HOMA index. In addition, the lack of a standardized international insulin assay makes difficult to compare results among studies [16]. A direct measure of IR was not available in this study. Therefore, it is not possible to evaluate whether HOMA2-IR is superior to HOMA1-IR with regard to insulin resistance. The diagnostic accuracy of HOMA indexes compared to a gold standard (sensitivity and specificity compared to the the hyperinsulinemic euglycemic clamp) was not examined in this study.

## 4. CONCLUSION

HOMA1-IR and HOMA2-IR are useful as diagnostic tools for IR and MS among Cuban-Americans in South Florida. Both indexes were associated with MS, however, HOMA2-IR showed a better association with MS components. Given the high prevalence of diabetes in this specific population, the use of a simple indirect tool to identify IR may allow for early prevention strategies.

## Figures and Tables

**Table 1.** Clinical and metabolic characteristics of the healthy and metabolic syndrome subjects

Characteristic	Healthy group Mean ± SD or median (IQR) (n=75)	Metabolic Syndrome Mean ± SD or median (IQR) (n=71)	*P*-value

Age (years)	60.99±11.98	65.04±9.65	.03
Female (%) BMI (kg/m^2^)	68.0	61.9	.45
	28.42±4.48	31.21 ±4.91	<.001
WC (cm)	94.99±9.22	105.11±12.85	<.001
SBP (mm Hg)	126.13±18.90	138.79±19.05	<.001
DBP (mm Hg)	79.36±8.81	85.92±9.63	<.001
TG (mg/dL)	105.08±37.96	191.41±100.50	<.001
TC (mg/dL)	205.09±36.33	212.83±50.60	.29
HDLC (mg/dL)	61.51±14.96	52.10±12.44	<.001
LDLC (mg/dL)	122.61±32.35	124.00±41.49	.82
FPG (mg/dL)	92.08±9.73	99.05±12.88	<.001
FPG ≥ 100 mg/dl (%)	0	36	<.001
Smoking yes (%)	3%	5%	.56
Insulin (mU/L)	10.41±5.01	14.04±6.27	<.001
HOMA1-IR	2.06 (1.3 to 3.1)	3.24 (2.3 to 4.5)	<.001^a^
HOMA2-IR	1.20 (0.9 to 1.7)	1.70 (1.2 to 2.3)	<.001^a^

BMI = body mass index, WC=weight circumference, SBP=systolic blood pressure, DBP=diastolic blood pressure, TG=triglycerides, TC=total cholesterol, HDLC=HDL-cholesterol, LDLC=LDL-cholesterol, FPG=fasting plasma glucose, HOMA=homeostatic model assessment, SD=standard deviation, IQR=interquartile range.

a Mann-Whitney test for HOMA1-IR and HOMA2-IR

**Table 2.** Cut-off points of HOMA1-IR and HOMA2-IR indexes to identify insulin resistance and metabolic syndrome

Index	Insulin resistance	Metabolic syndrome		
	
	Cut-off point^a^	cut-off point^b^	Sensitivity	Specificity

HOMA1-IR	3.95	2.98	63.4	73.3
HOMA2-IR	2.2	1.55	60.6	66.7

a the 90^th^ percentile in the healthy group

b optimal cut-off point from ROC analysis
